# Reproducibility of measurements of potential doubling time of tumour cells in the multicentre National Cancer Institute protocol T92-0045

**DOI:** 10.1038/sj.bjc.6690052

**Published:** 1999-01

**Authors:** G D Wilson, N Paschoud, J-J Pavy, K Weber, P Weber, B Dubray, P A Coucke

**Affiliations:** Gray Laboratory Cancer Research Trust, Mount Vernon Hospital, Northwood, Middlesex HA6 2JR, UK; Laboratoire de Cytométrie en Flux, Clinique La Source, Lausanne, Switzerland; Department of Radiotherapy, Centre Hospitalier Universitaire, Besançon, France; Oncologie Radiotherapeutique, Institut Curie, 26 Rue d'Ulm, Paris, 75248, France; Department of Radiotherapy, Centre Hospitalier Universitaire Vaudois, Lausanne, Switzerland

**Keywords:** proliferation, reproducibility, conventional radiotherapy, multicentre, flow cytometry

## Abstract

We compared the flow cytometric measurement and analysis of the potential doubling time (*T*_pot_) between three centres involved in the National Cancer Institute (NCI) protocol T92-0045. The primary purpose was to understand and minimize the variation within the measurement. A total of 102 specimens were selected at random from patients entered into the trial. Samples were prepared, stained, run and analysed in each centre and a single set of data analysed by all three centres. Analysis of the disc data set revealed that the measurement of labelling index (LI) was robust and reproducible. The estimation of duration of S-phase (*T*_s_) was subject to errors of profile interpretation, particularly DNA ploidy status, and analysis. The LI dominated the variation in *T*_pot_ such that the level of final agreement, after removal of outliers and ploidy agreement, reached correlation coefficients of 0.9. The sample data showed poor agreement within each of the components of the measurement. There was some improvement when ploidy was in agreement, but correlation coefficients failed to exceed values of 0.5 for *T*_pot_. The data suggest that observer-associated analysis of *T*_s_ and tissue processing and tumour heterogeneity were the major causes of variability in the *T*_pot_ measurement. The first two aspects can be standardized and minimized, but heterogeneity will remain a problem with biopsy techniques. © 1999 Cancer Research Campaign


British Journal of Cancer (1999) **79**(2), 323–332
© 1999 Cancer Research Campaign

### Reproducibility of measurements of potential doubling time of tumour cells in the multicentre National Cancer Institute protocol T92-0045

**GD Wilson[1], N Paschoud[2], J-J Pavy[3], K Weber[2], P Weber[2], B Dubray[4] and PA Coucke[5]**

[1]Gray Laboratory Cancer Research Trust, Mount Vernon Hospital, Northwood, Middlesex HA6 2JR, UK; [2]Laboratoire de Cytométrie en Flux, Clinique La Source, Lausanne, Switzerland; [3]Department of Radiotherapy, Centre Hospitalier Universitaire, Besançon, France; [4]Oncologie Radiotherapeutique, Institut Curie, 26 Rue d'Ulm, 75248 Paris, France; [5]Department of Radiotherapy, Centre Hospitalier Universitaire Vaudois, Lausanne, Switzerland

**Summary** We compared the flow cytometric measurement and analysis of the potential doubling time ($T_{pot}$) between three centres involved in the National Cancer Institute (NCI) protocol T92-0045. The primary purpose was to understand and minimize the variation within the measurement. A total of 102 specimens were selected at random from patients entered into the trial. Samples were prepared, stained, run and analysed in each centre and a single set of data analysed by all three centres. Analysis of the disc data set revealed that the measurement of labelling index (LI) was robust and reproducible. The estimation of duration of S-phase ($T_s$) was subject to errors of profile interpretation, particularly DNA ploidy status, and analysis. The LI dominated the variation in $T_{pot}$ such that the level of final agreement, after removal of outliers and ploidy agreement, reached correlation coefficients of 0.9. The sample data showed poor agreement within each of the components of the measurement. There was some improvement when ploidy was in agreement, but correlation coefficients failed to exceed values of 0.5 for $T_{pot}$. The data suggest that observer-associated analysis of $T_s$ and tissue processing and tumour heterogeneity were the major causes of variability in the $T_{pot}$ measurement. The first two aspects can be standardized and minimized, but heterogeneity will remain a problem with biopsy techniques.

**Keywords:** proliferation; reproducibility; conventional radiotherapy; multicentre; flow cytometry

Radiotherapy remains an effective treatment modality in cancer management, being administered to 50–60% of all patients with malignant disease. Improvements in the success of radiotherapy can be expected from better dose prescription and the rational use of altered fractionation schedules and radiomodifiers and by the selection of patients for the most appropriate treatment.

Proliferation of tumour clonogens during treatment is thought to be a major reason for the failure of conventional (6–7 weeks) fractionation schedules to cure some tumours (Withers et al, 1988; Fowler and Lindstrom, 1992). If tumours that are likely to undergo rapid repopulation could be identified prior to treatment, reducing the overall treatment time using accelerated fractionation schedules (Peters et al, 1988; Fowler, 1990; Dische et al, 1997; Horiot et al, 1997) might increase the probability of controlling them.

Currently, the use of the halogenated pyrimidines and flow cytometry (FCM) is considered to be the best method to measure proliferation rates in vivo as the technique requires only a single biopsy to measure the potential doubling time (Begg et al, 1985). However, evidence that the $T_{pot}$ measurement can predict the outcome of radiotherapy has still to be established unequivocally (Begg, 1995). The reasons for this are twofold. Firstly, many of the studies in which $T_{pot}$ measurements have been applied to radiotherapy patients suffer from small patient numbers (Begg et al, 1990; Bourhis et al, 1993; Corvo et al, 1993). Secondly, the accuracy and reproducibility of the $T_{pot}$ measurement may not be adequate.

In 1992, a cooperative was set up to address these two issues in a large multicentre study of conventional radiotherapy. The NCI-sponsored T92-0045 trial consists of 23 European centres, which set out to accrue 1000 patients in four tumour localizations (head and neck, rectum, uterine cervix and bronchus); all patients were treated by conventional radiotherapy and in all cases a pretreatment $T_{pot}$ measurement was made. To date, over 800 patients have been entered into the trial.

In this paper, we report on a study of 102 sequential specimens from 97 patients entered into the trial. Three laboratories have investigated aspects of sample preparation and analysis to establish where the major sources of variation exist. We discuss whether quality control guidelines and a consensus opinion can increase the reliability and clinical utility of the measurement.

*Received 5 March 1998*
*Revised 14 May 1998*
*Accepted 7 June 1998*

*Correspondence to:* GD Wilson

### MATERIALS AND METHODS

### Patients

Material for the study was selected on a consecutive basis, the only criterion being that there should be enough tumour material. A total of 102 specimens were processed and analysed in the three centres and a single set of disc data, from these same patients, was analysed in each centre. The specimens consisted of 25 cervix, 36 oral cavity, 35 rectum and six lung tumours originating from 11 of the participating centres. Each patient had received an intravenous injection of 100 mg m$^{-2}$ iododeoxyuridine (IdUrd) (NCI, Investigational Drugs Branch, Brussels) several hours prior to the surgical procedure. The median time between injection and surgery was 6.33 h (range 3–10 h, 5.83 and 7.0 h being the 25th and 75th quartiles).

### Sample preparation and staining

The specimens were fixed in ice-cold 70% ethanol for storage. Prior to the study, each centre agreed on a basic sample preparation and staining protocol. The fragments of tissue were minced using scissors and placed in 5 ml of 0.1 M hydrochloric acid containing 0.4 mg $ml^{-1}$ porcine trypsin (Sigma, St. Louis, MO, USA) and incubated at 37°C until the tissue was digested; this was typically between 45 and 60 min. The resultant nuclei suspension was filtered through 35-μm nylon mesh and centrifuged at 2000 r.p.m. for 5 min. The resulting pellet was resuspended in 2 ml of 2 M hydrochloric acid for 15 min to unwind DNA partially, allowing monoclonal antibody access to IdUrd. The samples were then washed twice with 5 ml of phosphate-buffered saline (PBS) to remove the acid and then resuspended in 0.5 ml of PNT (PBS containing 0.5% normal goat serum and 0.5% Tween-20) and 20 μl of mouse anti-5-bromo-2′-deoxyuridine (BrdUrd)/IdUrd monoclonal antibody (Dako). The tubes were incubated for 1 h at room temperature in the dark. After washing with PBS, the pellets were resuspended in 0.5 ml of PNT containing 20 μl of goat anti-mouse IgG (whole molecule) fluoroscein isothiocyanate (FITC) conjugate and incubated for 30 min at room temperature. After washing with PBS, the specimens were resuspended in 1 ml of PBS containing 10 μg $ml^{-1}$ propidium iodide.

### Flow cytometry

The samples were analysed on FACScan flow cytometers (Becton Dickinson, San Jose, CA, USA) in each centre. The DNA signal from propidium iodide was collected into the FL3 channel and cell doublets discriminated using the width and area signal. The FITC signal from IdUrd was collected on a logarithmic amplifier in the FL1 channel. At least 10 000 single events were collected.

### Data analysis

Data analysis consisted of three distinct procedures: the decision as to the ploidy status, the setting of the regions to detect IdUrd incorporation and the setting of the regions to measure $T_s$. The initial decision as to the ploidy status governed the regions that were used to assess the kinetic parameters. A tumour was considered diploid if only one stem line of cells could be observed. The decision as to whether a tumour was diploid or tetraploid was based on both the proportion of $G_2$/tetraploid $G_1$ cells and the presence of IdUrd labelling. If significant labelling, distributed in the expected pattern, was associated with a 4*n* to 8*n* population, it was considered tetraploid irrespective of the proportion of cells. If the proportion of diploid $G_2$ cells exceeded 15%, then tetraploidy was classified. The major $G_1$ peak classified aneuploid tumours, but polyploidy was noted. The decision as to whether a tumour was hypo- or hyperdiploid was based on which population contained proliferating cells, i.e. if the first $G_1$ peak showed proliferation this would be assumed to be the tumour population and the classification would be hypodiploid. In some tumours with near-diploid DNA, it was not possible to analyse the labelled populations separately and these represented a category of tumours that were classified as aneuploid but analysed as diploid.

Figure 1 shows the FCM profiles and regions required for analysing both a diploid and an aneuploid tumour. After a region (R1) was set for doublet discrimination on the FL3 area and width dot plot, a second region (R2) was set on the gated FL3 area and FL1 dot plot, to discriminate the IdUrd-labelled cells. The lower limit of this region was set by experience rather than using a control. The data for LI and $T_s$ were analysed from single-parameter DNA histograms generated from the total and the IdUrd-labelled cells only.

In diploid tumours, a total of four markers were set. On the total DNA profile, M1 and M2 identified the $G_1$ and $G_2$ populations, respectively, for analysis of $T_s$. On the IdUrd-labelled DNA profile, M3 and M4 marked the divided cells and those still moving through S-phase for correction of the LI and calculation of $T_s$ respectively. In aneuploid tumours, four further regions were set. M1 and M2 identified the diploid and aneuploid $G_1/G_0$ populations for calculation of the DNA index, M2 and M3 contained the $G_1$ and $G_2$ of the aneuploid population and M4 described the total number of aneuploid cells. In the IdUrd-labelled DNA profile, M5 and M6 were used to correct for cell division in the diploid and aneuploid population, M7 delineated the population still traversing S-phase for the calculation of $T_s$ and M8 measured the total number of aneuploid labelled cells.

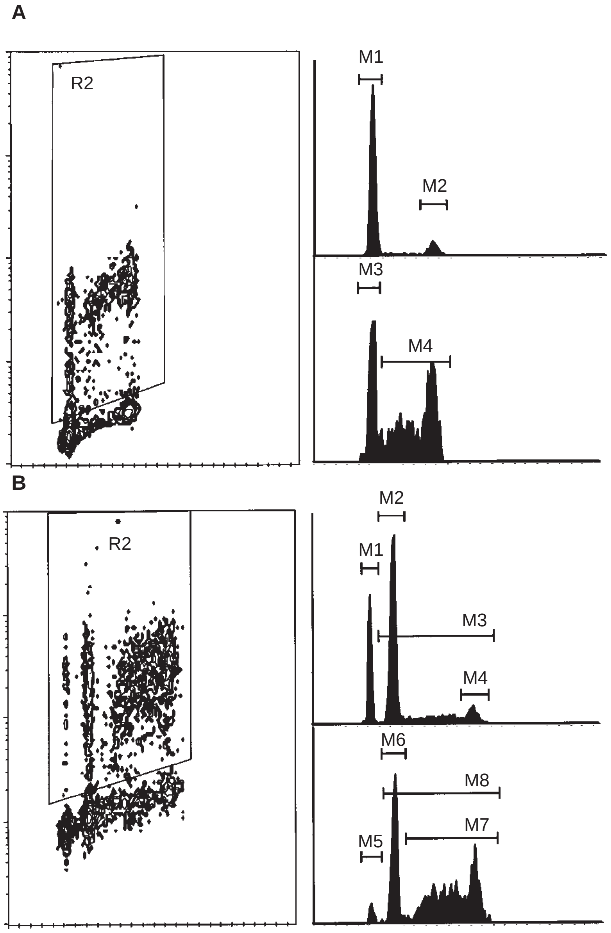

**Figure 1** Typical examples of a diploid (**A**) and aneuploid (**B**) flow cytometry profile. The dot plot (left) shows the region delineating BrdUrd-labelled cells. The histograms (right) show the DNA profile of all single nuclei (**A**) and of BrdUrd-labelled nuclei only. See text for description of markers

© Cancer Research Campaign 1999

### Calculation of DNA index, LI, $T_s$ and $T_{pot}$

All data were handled electronically; the numerical information was imported directly into an Excel spreadsheet from the FCM analysis program (PC Lysys).

The DNA index was calculated using the ratio of M2/M1 for aneuploid tumours. The total LI (TLI) was calculated making a correction for those cells that had divided between injection and biopsy. For diploid tumours, the calculation was as follows using the numbers of cells in each of the following regions:

$$\frac{\text{Total labelled cells} - \text{M3}/2}{\text{Total cells} - \text{M3}/2}$$

In aneuploid tumours the TLI was calculated as:

$$\frac{\text{Total labelled cells} - (\text{M5} + \text{M6})/2}{\text{Total cells} - (\text{M5} + \text{M6})/2}$$

In addition, the corrected LI could be calculated for the tumour cells only (ALI):

$$\frac{\text{M8} - \text{M6}/2}{\text{M4} - \text{M6}/2}$$

The $T_s$ was calculated using the relative movement method (Begg et al, 1985) in which the mean DNA content of the $G_1$ and $G_2$ populations and that of the IdUrd-labelled cells, yet to divide, are required to calculate a parameter termed the relative movement (RM). The regions used for this are shown below, with the corresponding regions for aneuploid tumours in parentheses.

$$\text{RM} = \frac{\text{M4(M7)} - \text{M1(M2)}}{\text{M2(M3)} - \text{M1(M2)}}$$

The $T_s$ was calculated from the RM using the original assumptions that the RM at time zero was 0.5 because of the uniform distribution of labelled cells throughout S-phase and that the value reaches 1.0 at a time equal to $T_s$ because of uniform progression of labelled cells through S. The $T_s$ was simply calculated from the relationship:

$$T_s = \frac{0.5 \times t \text{ (time between injection and biopsy)}}{\text{RM} - 0.5}$$

The $T_{pot}$ was computed from the $T_s$ and the LI using the formula of Steel (1977)

$$T_{pot} = \frac{\lambda \times T_s}{\text{LI}}$$

where λ is a correction factor for the age distribution of the population and was assumed to be 0.8.

### Statistical analysis

A variety of statistical tests were applied, including linear regression analysis, Spearman's rho, κ measure of agreement and Bland–Altman methods for assessing agreement (Bland and Altman, 1986). Statistical analyses were carried out using JMP (SAS Institute, Cary, NC, USA).

### RESULTS

### Initial characterization of the data set

Table 1 summarizes the cell kinetic parameters obtained for the complete set of samples in each of the three centres. The nomenclature was such that the first letter represented the centre where the sample was processed and the second letter indicated where the sample was analysed; thus, LG signifies that the sample was processed in Lausanne and analysed in the Gray Laboratory. Considerable variation was present within and between data sets. The most notable discrepancies were the differences between the median and mean values for $T_s$, indicating a non-symmetric distribution. This was particularly evident within the single data set analysed by all three centres in which the standard deviations were greater than those in samples processed separately in each centre. This variability was translated into the data for $T_{pot}$, which showed significant differences between the data sets.

The underlying reason for this result was the presence of spuriously high $T_s$ values within some of the disc analysis data sets and which were not reciprocated within the sample data set. In one specimen, an extreme value of 1432 h was calculated for $T_s$ in one centre in comparison with 14.1 and 61.1 h from the other two centres. This was a particularly difficult and confusing profile to analyse and, of the 102 specimens, there were 12 in which either one or two of the centres recorded $T_s$ values that were considered to be spuriously high (> 40 h). Censoring of these data resulted in better agreement between mean and median values for $T_s$ and $T_{pot}$ (Table 1) but was without effect on LI.

### Spearman rank correlation analysis

The true test of the $T_{pot}$ measurement is whether the estimated values are reproducible between different observers and different centres in the ranking of tumours as fast or slow. Table 2 demonstrates that the major discrepancies in the single data set reside within the calculation of $T_s$ as evidenced by the low correlation coefficients for both RM and $T_s$. The agreement in determining the total LI was consistently high between all observers. The agreement in calculating the aneuploid LI was less precise and, as will be discussed later, was primarily due to interpretation of the DNA profile. $T_{pot}$ relies on both the LI and $T_s$ and, as a result, the agreement for this parameter was intermediate among that found for its determinants. However, the agreement was improved considerably by censoring the specimens with outlying $T_s$ values. The level of concordance in $T_{pot}$ between the three centres resulted in correlation coefficients ranging from 0.78 to 0.86.

The correlation coefficients when samples were processed and analysed in different centres fell dramatically for all parameters. The loss of agreement might result from variability in sample processing, staining and running and from tumour heterogeneity. Consideration of the LI data would appear to suggest that the LI might be the major determinant. The level of agreement was better for the aneuploid LI than for the total LI, the reverse of the disc data. Within a specimen, it is likely that the LI of the tumour cells alone might be more consistent between different areas than the total LI, which includes stromal and infiltrating cells. The correlation for $T_s$ was only slightly worse than for the disc data. The combination of these two parameters resulted in correlation coefficients of 0.30–0.45 for $T_{pot}$ irrespective of whether the complete or restricted data sets were considered. The disc and

© Cancer Research Campaign 1999

Table 1 Summary of kinetic parameters obtained from each centre for all 102 specimens. The numbers in parentheses represent the data obtained from 90 patients excluding the censored $T_s$ data

| | LL | LB | LG | BB | GG |
|---|---|---|---|---|---|
| TLI | | | | | |
| Median | 0.094 | 0.093 | 0.093 | 0.105 | 0.073 |
| Mean | 0.104 | 0.110 | 0.102 | 0.118 | 0.083 |
| s.d. | 0.060 | 0.110 | 0.055 | 0.070 | 0.051 |
| ALI | | | | | |
| Median | 0.186 | 0.166 | 0.180 | 0.194 | 0.119 |
| Mean | 0.200 | 0.204 | 0.195 | 0.217 | 0.131 |
| s.d. | 0.103 | 0.158 | 0.098 | 0.131 | 0.076 |
| $T_s$ | | | | | |
| Median | 14.2 (13.4) | 13.7 (13.1) | 11.7 (11.5) | 11.3 (11.1) | 10.9 (10.7) |
| Mean | 20.0 (14.9) | 32.1 (14.8) | 14.0 (12.9) | 12.7 (11.8) | 11.8 (11.5) |
| s.d. | 22.6 (5.6) | 141.1 (6.0) | 10.4 (5.2) | 6.0 (3.4) | 4.4 (4.3) |
| $T_{pot}$ | | | | | |
| Median | 3.8 (3.6) | 4.2 (4.2) | 3.3 (3.4) | 2.9 (2.8) | 4.0 (4.1) |
| Mean | 5.8 (5.4) | 9.8 (6.7) | 4.3 (4.2) | 5.2 (4.4) | 5.2 (5.3) |
| s.d. | 6.1 (5.6) | 32.5 (10.4) | 3.2 (2.8) | 8.2 (5.4) | 4.0 (4.1) |

Table 2 Spearman's rank correlation coefficient analysis. Data are presented for all specimens and for 90 cases excluding the outlying $T_s$ values. All of the combinations were significantly correlated, except for the RM data for the comparison of BB and GG. In this data set, a correlation coefficient of 0.34 results in $P$-value < 0.0001

| Parameter | Sample | | | Disc | | | Disc/sample | |
|---|---|---|---|---|---|---|---|---|
| | LL BB | LL GG | BB GG | LL LB | LL LG | LB LG | LB BB | LG GG |
| RM (102) | 0.44 | 0.50 | 0.13 | 0.63 | 0.50 | 0.36 | 0.35 | 0.40 |
| (90) | 0.50 | 0.51 | 0.19 | 0.63 | 0.60 | 0.42 | 0.34 | 0.46 |
| TLI (102) | 0.50 | 0.49 | 0.53 | 0.94 | 0.95 | 0.95 | 0.48 | 0.48 |
| (90) | 0.53 | 0.50 | 0.60 | 0.94 | 0.96 | 0.96 | 0.50 | 0.50 |
| ALI (102) | 0.65 | 0.55 | 0.59 | 0.91 | 0.83 | 0.87 | 0.61 | 0.58 |
| (90) | 0.68 | 0.49 | 0.61 | 0.91 | 0.85 | 0.86 | 0.64 | 0.58 |
| $T_s$ (102) | 0.49 | 0.50 | 0.37 | 0.69 | 0.50 | 0.46 | 0.43 | 0.55 |
| (90) | 0.50 | 0.47 | 0.40 | 0.63 | 0.61 | 0.51 | 0.44 | 0.63 |
| $T_{pot}$ (102) | 0.36 | 0.30 | 0.36 | 0.82 | 0.61 | 0.59 | 0.49 | 0.24 |
| (90) | 0.45 | 0.31 | 0.43 | 0.84 | 0.86 | 0.78 | 0.47 | 0.30 |

sample comparison between the Besancon and Gray Laboratory data sets resulted in correlation coefficients similar to those seen within the sample comparison.

### Kappa statistic analysis

The kappa correlation (κ) was chosen to measure the degree of agreement between samples. In this analysis, each parameter was classified according to whether it was above or below the median value for each individual data set, and the results for LI, $T_s$ and $T_{pot}$ are shown in Table 3. The data reiterate the findings of the Spearman's analysis. In the disc-only data, the level of agreement in TLI was excellent (> 0.8), $T_{pot}$ was in the good category (0.61–0.8) and the $T_s$ values fell into the moderate category of agreement (0.41–0.6). In the sample and the disc–sample data, the majority of κ values fell below 0.5, which indicated poor agreement.

### Bland–Altman analysis of agreement

The level of agreement was assessed using the procedure of Bland and Altman. The data are presented in Table 4 for TLI, $T_s$ and $T_{pot}$ for the restricted data set of 90 patients. The mean difference gives an indication of measuring bias in each data set and centre and two standard deviations defines the limits within which the differences would be expected to be found. Within the complete (data not shown) and censored data set, the TLI shows excellent agreement in the disc data, with mean differences of less than 0.1%. The 95% confidence intervals on the mean difference were no more than ± 0.3% around the mean value. The majority of differences would be found within ± 4.0% of the mean. However, within the sample and sample/disc data sets, the mean difference ranges from –2.0% to 3.9% and the standard deviations increased to between 5% and 6.0%. This would result in wide expected limits of agreement of ± 10–12% around the mean.

 © Cancer Research Campaign 1999

**Table 3** Kappa correlation analysis using the median value as a cut-off for each parameter in each data set

| Parameter | Sample | | | Disc | | | Disc/sample | |
|---|---|---|---|---|---|---|---|---|
| | LL BB | LL GG | BB GG | LL LB | LL LG | LB LG | LB BB | LG GG |
| TLI (102) | 0.47 | 0.33 | 0.45 | 0.92 | 0.92 | 0.92 | 0.45 | 0.29 |
| (90) | 0.38 | 0.33 | 0.38 | 0.82 | 0.82 | 0.96 | 0.29 | 0.33 |
| $T_s$ (102) | 0.49 | 0.33 | 0.29 | 0.65 | 0.45 | 0.49 | 0.41 | 0.45 |
| (90) | 0.51 | 0.33 | 0.38 | 0.69 | 0.56 | 0.57 | 0.42 | 0.51 |
| $T_{pot}$ (102) | 0.37 | 0.18 | 0.22 | 0.81 | 0.65 | 0.69 | 0.37 | 0.14 |
| (90) | 0.37 | 0.22 | 0.22 | 0.78 | 0.73 | 0.78 | 0.38 | 0.38 |

**Table 4** Bland–Altman analysis of the agreement between centres for the restricted data set (90) specimens. The data represent the mean difference and standard deviation for each pair of observer and centre combinations

| Centres | TLI (%) | | $T_s$ (h) | | $T_{pot}$ (days) | |
|---|---|---|---|---|---|---|
| | Mean | s.d. | Mean | s.d | Mean | s.d |
| BBGG | 3.9 | 5.7 | 0.25 | 4.16 | –0.83 | 6.29 |
| BBLL | 1.9 | 6.0 | –3.05 | 5.16 | –0.95 | 6.60 |
| GGLL | –2.0 | 5.4 | –3.30 | 4.93 | –0.12 | 6.36 |
| LBLG | –0.1 | 1.6 | 1.94 | 6.61 | 2.48 | 8.71 |
| LBLL | –0.1 | 2.0 | 0.0002 | 5.04 | 1.34 | 5.19 |
| LGLL | –0.03 | 1.4 | –1.94 | 4.44 | –1.14 | 4.09 |
| BBLB | 2.0 | 6.2 | –3.05 | 5.64 | –2.29 | 10.37 |
| GGLG | –2.0 | 5.1 | –1.37 | 3.88 | 1.03 | 4.03 |

The data for $T_s$ indicate variable agreement in both the disc and sample data, with the mean differences ranging from –3.3 to 1.9 h. There was a trend for both Besancon and the Gray Laboratory to produce lower $T_s$ values than Lausanne. The important parameter is the standard deviation, which varies between 4 and 6 h for each combination, indicating that observers or centres could differ by as much as ± 12 h from the mean value. The variability in $T_s$ was translated into differences in $T_{pot}$. Within the restricted data set, the mean differences were reduced to ± 2 days and the limits of agreement ranged from 4 to 10 days around the mean.

### The influence of DNA ploidy

The analysis of $T_{pot}$ is dependent on the classification and interpretation of the DNA profile. In the complete data set, 34 tumours were classified as diploid and 20 were classified as aneuploid (with the same DNA index) by all observers and centres; these represent 53% of the specimens. A further five specimens were uniformly classified as aneuploid but with some discrepancy in the DNA index values. In a further 25 specimens, four out of the five combinations agreed on either an aneuploid (18 cases) or a diploid (seven cases) profile. The majority of these discrepancies (22 cases) arose within the sample data. To examine the influence of DNA ploidy, the disc data and sample data sets were analysed separately to establish which factors were attributable to observer variation alone and which to observer and sampling variation.

### *Disc data set*

Forty-two specimens were agreed as diploid and 41 were uniformly classified as aneuploid with similar DNA indices, representing 81% of the data set. The discrepant tumours were eight in which two observers classified the specimen as aneuploid and the other reported a diploid profile and six in which two diploid values were recorded and one aneuploid. One specimen had three different aneuploid values, three specimens had two similar aneuploid indices and one discrepant and one specimen was classified as diploid, hyperdiploid and hypodiploid. In the restricted data set (90 specimens), 42 specimens were diploid, 34 were uniformly aneuploid, six were classified as aneuploid by two observers and diploid by the other and five were classified as diploid by two observers and aneuploid by the other. Only three specimens did not fit into these classifications. It was noted that the 12 specimens excluded from the censored data set were either aneuploid or discrepant cases.

Table 5 summarizes the improvement in agreement when consensus on ploidy was reached. The agreement in TLI was further increased to a coefficient of, on average, 0.97 while ALI coefficients exceeded 0.9. There was a significant increase in agreement in $T_s$ compared with the data in Table 2; the coefficients reached 0.8 for diploid tumours and 0.6–0.7 for aneuploid tumours. These improvements in agreement in LI and $T_s$ were translated into an increase in the correlation coefficients for $T_{pot}$, with values of 0.9 overall in the restricted data set. Again, agreement was better in diploid tumours (0.94) than that obtained in aneuploid specimens.

### *Sample data*

Ploidy agreement in the sample data set was worse than that found within the disc data set. In the 102 samples, 38 were classified as diploid by all three centres but only 21 were aneuploid with the same DNA index; this represents 58% of specimens. In a further 11 specimens, two diploid and one aneuploid classifications were recorded and, in 18 cases, two similar aneuploid values and one diploid were obtained. The rest consisted of seven cases in which there were two different aneuploid values accompanying one diploid, five cases with two similar aneuploid values with a different aneuploid DNA index and two cases with three different aneuploid indices.

Table 6 summarizes the correlation data obtained after ploidy agreement in the sample data. It can be seen (in comparison with Table 2) that there is some improvement in the correlation coefficients for TLI, $T_s$ and $T_{pot}$. This result was more evident in the complete data set rather than in the censored data set, as most of the censored data were due to aberrations in the disc data. Unlike the disc data (Table 5), the improvement in correlation for $T_{pot}$ was

*© Cancer Research Campaign 1999*

**Table 5** Spearman's correlation analysis of disc specimens in which there was consensus in ploidy classification. 'All' refers to that which were uniformly classified as diploid or aneuploid with the same DNA index and 'dip' and 'aneu' refer to the subgroups of diploid or aneuploid tumours only

| Parameter | LB vs LG | | | LB vs LL | | | LG vs LL | | |
|---|---|---|---|---|---|---|---|---|---|
| | All | Aneu | Dip | All | Aneu | Dip | All | Aneu | Dip |
| TLI (102) | 0.97 | 0.96 | 0.96 | 0.96 | 0.94 | 0.98 | 0.96 | 0.95 | 0.97 |
| (90) | 0.97 | 0.98 | | 0.97 | 0.95 | | 0.97 | 0.97 | |
| ALI (102) | | 0.97 | | | 0.90 | | | 0.89 | |
| (90) | | 0.98 | | | 0.90 | | | 0.92 | |
| $T_s$ (102) | 0.59 | 0.36 | 0.79 | 0.80 | 0.74 | 0.83 | 0.64 | 0.37 | 0.77 |
| (90) | 0.67 | 0.58 | | 0.77 | 0.70 | | 0.75 | 0.71 | |
| $T_{pot}$ (102) | 0.73 | 0.48 | 0.94 | 0.84 | 0.71 | 0.93 | 0.74 | 0.56 | 0.94 |
| (90) | 0.88 | 0.72 | | 0.90 | 0.76 | | 0.93 | 0.88 | |

**Table 6** Spearman's correlation analysis of sample specimens in which there was consensus in ploidy classification. 'All' refers to tumours that were uniformly classified as diploid or aneuploid with the same DNA index and 'dip' and 'aneu' refer to the subgroups of diploid or aneuploid tumours only

| Parameter | BB vs GG | | | BB vs LL | | | GG vs LL | | |
|---|---|---|---|---|---|---|---|---|---|
| | All | Aneu | Dip | All | Aneu | Dip | All | Aneu | Dip |
| TLI (102) | 0.66 | 0.69 | 0.69 | 0.63 | 0.66 | 0.58 | 0.63 | 0.68 | 0.63 |
| (90) | 0.60 | 0.57 | 0.68 | 0.53 | 0.50 | 0.55 | 0.50 | 0.42 | 0.63 |
| ALI (102) | | 0.68 | | | 0.68 | | | 0.71 | |
| (90) | | 0.61 | | | 0.68 | | | 0.49 | |
| $T_s$ (102) | 0.42 | 0.38 | 0.54 | 0.57 | 0.45 | 0.63 | 0.69 | 0.42 | 0.69 |
| (90) | 0.40 | 0.31 | 0.64 | 0.51 | 0.47 | 0.64 | 0.50 | 0.33 | 0.71 |
| $T_{pot}$ (102) | 0.53 | 0.55 | 0.55 | 0.59 | 0.66 | 0.52 | 0.49 | 0.58 | 0.52 |
| (90) | 0.43 | 0.40 | 0.54 | 0.45 | 0.42 | 0.51 | 0.31 | 0.26 | 0.52 |

not superior in diploid compared with aneuploid tumours, although the correlation coefficients for $T_s$ were superior in the diploid group. The correlation coefficients for TLI, $T_s$ and $T_{pot}$ were not dissimilar to each other, with average values ranging from 0.5 to 0.7, but agreement was generally better in TLI than in $T_s$.

### Proliferative classification using cut-off values

The ultimate requirement of the $T_{pot}$ measurement will be to classify tumours as fast or slow with some degree of certainty. In this analysis, the LG data were considered to be the 'true' data and the others compared with this. Figure 2 shows the data plotted as a ratio of the median LG value (3.44 days) for the restricted data set. The data in the upper left and lower right quadrants indicate the misclassified measurements. LL would wrongly classify 10% as fast and 12% as slow; for LB it would be 18% and 8%, GG 22% and 9% and BB 4% and 9%.

### DISCUSSION

In order to use a FCM measurement in clinical practice for the potential selection of patients for more appropriate treatment schedules, the sources of variation within the technique need to be understood and minimized. In common with all FCM-based methods there will be variation among centres associated with differences in instrumentation and laboratory techniques. Some of these can be minimized by using the same model of flow cytometer, agreed machine set-up and standardized laboratory procedures. This will leave the main source of variation to be attributable to what has been termed the interaction component. This comprises sample heterogeneity and inconsistencies in sample preparation, staining and analysis. If $T_{pot}$ values are to be meaningful and reproducible within and between laboratories, then this variation must be eliminated or minimized otherwise the technique will not be transportable or the data interchangeable.

The measurement of $T_{pot}$ from a single sample represents a relatively complex FCM procedure, many aspects of which have the potential to introduce variation (Terry and Peters, 1995). These include the sample itself, the tissue digestion with pepsin, the staining procedure (particularly the denaturation step), the interpretation of the DNA profile and the region setting for relative movement and labelling index analysis. Against this background, either the methodology must be robust or the tolerance limits of the measured parameter must be wide enough to permit some variation without compromising the clinical significance of the ultimate value. The NCI T92–0045 study was designed to address these two issues firstly with the comparisons reported here and, secondly, with the ultimate application of the measured values, with detailed knowledge of their variation, to the clinical data.

$T_{pot}$ reproducibility has been the subject of three previous reports (Wilson et al, 1993*a*; Haustermans et al, 1995; Tsang et al,

 © Cancer Research Campaign 1999

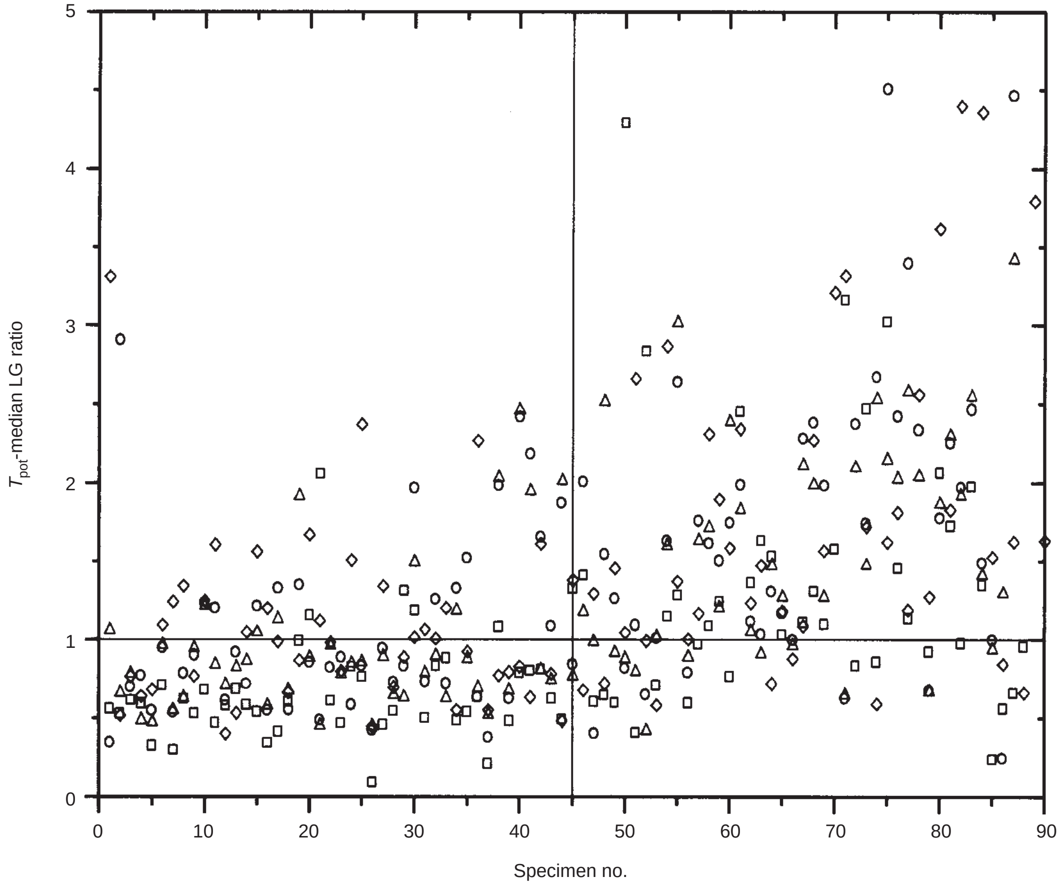

**Figure 2** Comparison of $T_{pot}$ values obtained from each centre and observer. The data are expressed as the ratio of the median value (3.44 days) from the LG restricted data set. The quadrants segregate the data into those which agree on a fast or slow classification (lower left and upper right) and those which disagree (upper right and lower left). The symbols represent BB (□), GG (◇), LB (○) and LL (Δ)

1995), which were restricted to two-centre analysis. The same general conclusions were reached in these studies, namely that the estimation of $T_s$ is the major source of analytical variation and that sample processing and tumour heterogeneity account for the variation between centres.

In this present multicentre study, we found the measurement of LI to be robust and reproducible in the disc data, with Bland–Altman analysis revealing no evidence of any systematic errors, as was found in one of the previous studies (Haustermans et al, 1995). Correlation analysis, both Spearman's and kappa, showed a high level of agreement among observers. Concordance of absolute values was extremely high, with linear regression analysis resulting in slopes of greater than 0.9 and intercepts of less than 0.01 (data not shown) for all three combinations. Indeed, if the LI was used to rank tumours according to their proliferative characteristics, only two specimens would be classified wrongly as fast and two as slow using this data set. The measurement of LI is a relatively simple procedure requiring only two regions. However, the first region, which delineates the IdUrd-labelled cells, is set subjectively and has received some criticism and discussion (White and Terry, 1992). The data in this study would suggest that fitting a distribution to the unlabelled $G_1$ and $G_2$ populations, in the IdUrd dot plot, and using standard deviations to set the lower limit of detection is unwarranted.

The estimation of $T_s$ was problematical. The analysis depends on the same initial region to delineate the IdUrd-labelled cells (which has been shown above to be reproducible) and a further three regions to measure the mean DNA content of $G_1$ and $G_2$ and of the IdUrd-labelled cells which have not divided. This should be a relatively straightforward procedure (as described in Figure 1) but is subject to the complexity of the IdUrd–DNA distribution. Twelve specimens were classified as outliers with the common feature of a $T_s$ value that was considered unreasonable, either too long (11 specimens) or too short (one specimen). All 12 specimens were classified as aneuploid by one or more observers, and 8 of the 12 specimens had discrepancies in the ploidy value. The majority of these specimens (11 cases) had low RM values of between 0.5 and 0.6 caused by multiploid DNA and the selection of the wrong $G_1$ or $G_2$ peak in the DNA profile for the RM calculation. It has been suggested that the ratio of $G_2$ to $G_1$ should be considered constant such that only the mean $G_1$ need be calculated (Begg et al, 1988). This procedure improves the correlation in the $T_s$ in the disc data but is without effect in the sample data.

The inconsistencies in region setting were highlighted by the section dealing with concordance in DNA ploidy prior to evaluation of the $T_s$. Indeed, even when all observers agreed that the profile was diploid, the correlations in $T_s$ failed to reach a value greater than 0.8. Analysis of the raw data in those diploid tumours that failed to correlate revealed that there was very good agreement in the mean values for the $G_1$ population but discrepancies in the measured mean DNA values for $G_2$ and the region defining the IdUrd-labelled cohort. The discrepancy in $G_2$ can arise from the tightness of the computer-generated region, which is dependent on the definition of the peak. The RM region is dependent on the tightness of the $G_1$ region and whether or not it has been set juxtaposed to this region (see Figure 1).

In aneuploid tumours that were ascribed the same DNA index by all observers, the correlation coefficients reached only 0.6 to 0.7 for $T_s$. The underlying reason for this was again variation in the region delineating the IdUrd-labelled cohort and to a lesser extent the $G_2$ population. The discrepancies with the former arose because of the presence of the diploid S and $G_2$ populations within the RM window. In some instances, the observers had attempted to eliminate them from the measurement by setting the lower limit of the analysis window to the right-hand side of the diploid $G_2$ rather than juxtaposed to the aneuploid $G_1$. This procedure is acceptable only if the IdUrd-labelled cohort of the aneuploid population has clearly progressed through S-phase, such that it is distinguishable from the diploid labelled population.

Although $T_s$ was the dominant feature in introducing variability into the agreement in the $T_{pot}$ measurement, the LI determines the extent of intrinsic variation in $T_{pot}$. This is primarily due to the broader distribution of potential values (40-fold variation) for LI compared with $T_s$ (eight-fold variation). To some extent, the reproducibility of LI overcomes the inherent problems within the $T_s$ estimation to produce excellent correlation coefficients for $T_{pot}$ once outliers and ploidy agreement are taken into account (0.9 or greater). In the disc data set, this cohort represented 75% of the original number of specimens analysed.

The sample data presents problems that have wider implications for the utility of $T_{pot}$ as a predictive measurement. The sample data were subject to all forms of potential variation and introduced the component due to sample processing and tumour heterogeneity. Tables 2 and 6 demonstrate that the correlation between all three centres is drastically reduced, compared with the disc data, for each of the parameters in the $T_{pot}$ measurement. Correlation analysis yielded values of no greater than 0.5–0.6 after accounting for ploidy differences; this comparison also introduced the greatest errors in the previous two reports of $T_{pot}$ reproducibility (Wilson et al, 1993*a*; Haustermans et al, 1995).

It was not possible to design this study to distinguish between sample processing and tumour heterogeneity because of the limitations of tissue. Clues from the data suggest that heterogeneity may be the dominant feature and thus a common problem to all biopsy-based measurements. In particular, the correlation for aneuploid LI exceeded that for total LI within the sample data, which is the converse of the disc data. The explanation for this reversal may reside within the consideration of genotypic and phenotypic heterogeneity within solid tumours (Shackney and Shankey, 1995). Tumour growth is determined by proliferation, differentiation and cell death, each of which is genetically controlled but subject to microenviromental stimuli such as nutrient and oxygen deprivation. Proliferative heterogeneity between samples from the same tumour will certainly arise as a function of differentiation, tumour growth pattern, host cell infiltration and vascular perfusion. Each of these will cause areas of microregional variation in the percentage of proliferating and non-proliferating cells and in the ratio of tumour cells to normal cells. The aneuploid LI is subject to the genotypic and phenotypic variability induced by differentiation, growth patterns and tumour perfusion. However, the total LI reflects the influence of both normal and tumour cells and may be also affected, to a greater extent, by heterogeneity due to variation in host cell content.

The mean intratumoral coefficient of variation (CV) of the three samples processed and analysed in each centre was 49% for $T_{pot}$. This was less than the 63% reported in the largest study of intratumour variability in $T_{pot}$ (Wilson et al, 1993*b*) in which six biopsies from 30 colorectal tumours were studied. In the colorectal study, CVs of 28% and 36% were reported for LI and $T_s$ compared with 38% and 27% in this present study. These data demonstrate that both $T_s$ and LI are important variables and can introduce variability into the ultimate $T_{pot}$ measurement. As has been found in other studies (Begg et al, 1988; Bennett et al, 1992; Wilson et al, 1993*a*), the intertumour variation in $T_{pot}$, 127% in this present study, far outweighs the intratumour variability. This indicates that, despite heterogeneity, proliferation differences between tumours should be detectable.

This present study demonstrates that significant differences exist between laboratories in the measurement of $T_{pot}$ and that these could result in misclassification of tumours as fast or slow by one or other centre. The following recommendations can minimize the analytical and processing errors but heterogeneity is inherent to the biology of individual tumours.

(1) Dissociation of the specimen into nuclei can introduce potential errors, particularly if underdigested (Terry and Peters, 1995). It is important to maximize the yield, in individual tumours, to increase the chance of obtaining a representative sample. The timing of dissociation should be adjusted for individual specimen needs, and it is not satisfactory to use a constant time interval for all tumours.

(2) Staining procedures should be standardized to that recommended by a laboratory with experience and the quality assessed by intercomparison of samples.

(3) Machine conditions should be standardized, although this will be different for different machines. Doublet discrimination should be used, the FITC signal should be collected with log amplification and at least 10 000 events should be recorded. The last is only a guideline as, for each sample, a significant number of events in the regions of interest need to be collected.

(4) Interpretation of the DNA profile should follow the guidelines already suggested (Shackney et al, 1993). Aneuploid populations should not be considered if they represent < 5% of the specimen unless the IdUrd staining can aid in their identification. All specimens of near-diploid DNA should be regarded as hyperdiploid unless the proliferating cells are clearly seen to emanate from the peak with less DNA. In near-diploid DNA profiles it will not always be possible to analyse the aneuploid population separately and all tumours should be analysed as a single population.

(5) In tumours with multiple DNA peaks, the choice of which aneuploid population to analyse should be based firstly on the magnitude of the population and, secondly, on the distribution of IdUrd labelling. The largest population would normally be analysed unless more proliferation was associated with a minor peak; this may well be the evolving clone of the tumour. The $G_1/G_2$ ratio should always be checked to ensure that regions have been set around the appropriate population.

© Cancer Research Campaign 1999

(6) $T_{pot}$ should not be analysed in profiles that show no evidence of cell division in the time between injection and biopsy. This may well be a function of the time interval, which should not be less than 4 h.
(7) $T_{pot}$ should not be analysed in profiles in which the region of interest is significantly impeded by overlapping cell populations from the diploid component or other aneuploid clones. It is not possible to set strict guidelines to this potential artefact, and this should be gained by experience or with reference to profiles obtained by other expert researchers.
(8) The regions to measure LI and $T_s$ should be as described in Figure 1, with the tightness of the $G_1$ and $G_2$ regions being determined by the CV of the corresponding peaks. Cell cycle analysis of the DNA profile may represent a better method of assessing the mean DNA contents of $G_1$ and $G_2$ and should be carried out if possible. The RM window should be set juxtaposed to the $G_1$ region, except in aneuploid tumours in which the labelled population has clearly progressed through its S-phase to a value greater than the diploid $G_2$.
(9) The transcription of data should be electronic to avoid errors.
(10) The calculation of $T_{pot}$ has been carried out using the Begg algorithm in this study, but several other alternatives give different absolute values of $T_{pot}$ but similar ranking of tumours (White et al, 1990). The clinical significance of each derivation of $T_{pot}$ will be assessed in the final correlation of the measurement with clinical outcome in T92-0045 trial.
(11) The question of heterogeneity can only be tackled by obtaining a large specimen and assessing at least two, and more if possible, areas from the sample. This may not always be possible and the quality of the nuclei suspension becomes crucial to ensure that it is representative. Whatever the sample, it should always be accompanied by histological assessment to ensure that a tumour is present and to give insight into the composition of the specimen.

The overall conclusion from this intercomparison is that agreement can be reached on the measurement of $T_{pot}$ in at least 75% of specimens by different observers analysing the same data. However, in a multicentre study, processing and analysis should be restricted to a single centre of excellence for the most consistent results. This does not preclude the interinstitutional comparison of results as long as the potential errors are clearly understood.

### ACKNOWLEDGEMENTS

We wish to thank the following as representatives of the participating centres in the trial: Dr J Bernier, Ospedale San Giovanni Leclerc, Radioterapia, CH-6500 Bellinzona; Dr TD Nguyen, Insitut J. Godinot, Service de Radiothérapie, 1 av. Général Koenig, F-51056 Reims; Dr PH Lagarde, Fondation Bergonie, Service de Radiothérapie, 180, Rue Saint-Genès, F-33076 Bordeaux Cedex; Dr K Beer, Inselspital, Klinik Für Radio-Onkologie, 3010 Bern; Dr PH Maingon, Centre Georges-Francois, Service de Radiothérapie, 1, Rue du Professeur Marion, F-21034 Dijon; Dr R Miralbell, Hopital Cantonal Universitaire de Geneve, Service de Radio-Oncologie, CH-1211 Geneve; Dr X Sun, Centre Hospitalier A. Boulloche, Service d'Oncologie et Radiothérapie, 2, rue du Dr Flamand, F-25209 Montbeliard; Dr G Storme, Vrije Universiteit, Oncologisch Centrum, Laarbeeklaan 101, B-1090 Bruxelles; Dr P Mere, Centre Leon Berard, Service de Radiothérapie, 28, Rue Laënnec, F-69373 Lyon Cedex 08; Dr J-P Gerard, Centre Hospitalier, Service de Radiothérapie–Oncologie, Pavillon 1F, F-69495 Pierre-Benite Cedex; Dr W Rogowski, Gdansk School of Medicine, Department of Oncology and Radiotherapy, U1. 7 Debinki St, P-80-211 Gdansk; Dr K Beer, Kantonsspital Basel, Hno Poliklinik, Petersgraben, CH-4031 Basel, Dr A Zouhair, Centre Universitaire Hospitalier Vaudois, Rue du Bugnon 46, CH-1011 Lausanne.

### REFERENCES

Begg AC (1995) The clinical status of Tpot as a predictor? Or why no tempest in the Tpot! *Int J Radiat Oncol Biol Phys* **32**: 1539–1541
Begg AC, Hofland I, Moonen L, Bartelink H, Schraub S, Bontemps P, Le Fir R, Van den Bogaert W, Caspers R, van Glabbeke M and Horiot J-C (1990) The predictive value of cell kinetic measurements in a European trial of accelerated fractionation in advanced head and neck tumours: an interim report. *Int J Radiat Oncol Biol Phys* **19**: 1449–1453
Begg AC, Moonen L, Hofland I, Dessing M and Bartelink H (1988) Human tumour cell kinetics using a monoclonal antibody against iododeoxyuridine; intratumour sampling variations. *Radiother Oncol* **11**: 337–347
Begg AC, McNally NJ, Shrieve DC and Karcher HA (1985) A method to measure the DNA synthesis and the potential doubling time from a single sample. *Cytometry* **6**: 620–626
Bennett MH, Wilson GD, Dische S, Saunders MI, Martindale CA and Robinson BM (1992) Tumour proliferation assessed by combined histological and flow cytometric analysis: implications for therapy in squamous cell carcinoma in the head and neck. *Br J Cancer* **65**: 870–878
Bland MJ and Altman DG (1986) Statistical methods for assessing agreement between two methods of clinical measurement. *Lancet* **i**: 307–310
Bourhis J, Wilson G, Wibault P, Bosq J, Chauvaudra N, Janot F, Luboinski B, Eschwege F and Malaise EP (1993) *In vivo* measurement of potential doubling time by flow cytometry in oropharyngeal cancer treated by conventional radiotherapy. *Int J Radiat Oncol Biol Phys* **26**: 793–799
Corvo R, Giaretti W, Sanguinsti G, Geido E, Orecchia R, Barra S, Margarino G, Bacigalupo A and Vitale V (1993) Potential doubling time in head and neck tumours treated by primary radiotherapy – preliminary evidence for a prognostic significance in local control. *Int J Radiat Oncol Biol Phys* **27**: 1165–1172
Dische S, Saunders M, Barrett A, Harvey A, Gibson D and Parmar M (1997) A randomised multicentre trial of CHART versus conventional radiotherapy in head and neck cancer. *Radiother Oncol* **44**: 123–136
Fowler JF (1990) How worthwhile are short schedules in radiotherapy? A series of exploratory calculations. *Radiother Oncol* **18**: 165–181
Fowler JF and Lindstrom M (1992) Loss of local control with prolongation in radiotherapy. *Int J Radiat Oncol Biol Phys* **23**: 457–467
Haustermans K, Hofland I, Pottie G, Ramaekers M and Begg AC (1995) Can measurements of potential doubling time (Tpot) be compared between laboratories? A quality control study. *Cytometry* **19**: 154–163
Horiot JC, Bontemps P, van den Bogaert W, Le Fur R, van den Weijngaert D, Bolla M, Bernier J, Lusinchi A, Stuschke M, Lopez-Torrecilla J, Begg AC, Pierart M and Collette L (1997) Accelerated fractionation (AF) compared to conventional fractionation (CF) improves loco-regional control in the radiotherapy of advanced head and neck cancers: results of the EORTC 22851 randomized trial. *Radiother Oncol* **44**: 111–121
Peters LJ, Ang KK and Thames HJ (1988) Accelerated fractionation in the radiation treatment of head and neck cancer. A critical comparison of different strategies. *Acta Oncol* **27**: 185–194
Shackney SE and Shankey TV (1995) Genetic and phenotypic heterogeneity of human malignancies: Finding order in chaos. *Cytometry* **21**: 2–5
Shankey TV, Rabinovitch PS, Bagwell B, Bauer KB, Duque RE, Hedley DW, Mayall BH, Wheeless L and Cox C (1993) Guidelines for implementation of clinical DNA cytometry. *Cytometry* **14**: 472–477
Steel GG (1977) *Growth Kinetics of Tumours*. Clarendon Press: Oxford
Terry NHA and Peters LJ (1995) The predictive value of tumour cell kinetic parameters in radiotherapy: Considerations regarding data production and analysis. *J Clin Oncol* **13**: 1833–1836
Tsang RW, Fyles AW, Kirkbride P, Levin W, Manchul LA, Rawlings GA, Banerjee M, Pintillie M and Wilson GD (1995) Proliferation measurements with flow cytometry Tpot in cancer of the uterine cervix: preliminary results. *Int J Radiat Oncol Biol Phys* **32**: 1319–1329

© Cancer Research Campaign 1999

Wheeless LL, Coon JS, Cox C, Deitch AD, deVere White RW and Fradet Y (1991) Precision of DNA flow cytometry in inter-institutional analyses. *Cytometry* **12**: 405–412

Wheeless LL, Coon JS, Cox C, Deitch AD, deVere White RW and Koss LG (1989) Measurement variability in DNA flow cytometry of replicate samples. *Cytometry* **10**: 731–738

White RA and Terry NHA (1992) A quantitative method for evaluating bivariate flow cytometric data obtained using monoclonal antibodies to bromodeoxyuridine. *Cytometry* **13**: 490–495

White RA, Terry NHA, Meistrich ML and Calkins, DP (1990) An improved method for computing the potential doubling time using monoclonal antibodies to bromodeoxyuridine. *Cytometry* **11**: 314–317

Wilson GD, McNally NJ, Dische S, Saunders MI, Des Rochers C, Lewis AA and Bennett MH (1988) Measurement of cell kinetics in human tumours *in vivo* using bromodeoxyuridine incorporation and flow cytometry. *Br J Cancer* **58**: 423–431

Wilson MS, West CM, Wilson GD, Roberts SA, James RD and Schofield PF (1993*a*) An assessment of the reliability and reproducibility of measurement of potential doubling times (Tpot) in human colorectal cancers. *Br J Cancer* **67**: 754–759

Wilson MS, West CML, Wilson GD, Roberts SA, James RD and Schofield PF (1993*b*) Intra-tumoural heterogeneity of tumour potential doubling times ($T_{pot}$) in colorectal cancer. *Br J Cancer* **68**: 501–506

Withers HR, Taylor JMG and Maciejewski B (1988) The hazard of accelerated tumour clonogen repopulation during radiotherapy. *Acta Oncol* **27**: 131–146

 *© Cancer Research Campaign 1999*
